# The effects of thiopurine therapy on health-related quality of life in Inflammatory Bowel Disease patients

**DOI:** 10.1186/1471-230X-10-26

**Published:** 2010-03-02

**Authors:** Guillermo Bastida, Pilar Nos, Mariam Aguas, Belén Beltrán, Marisa Iborra, Vicente Ortiz, Vicente Garrigues, Rafael Estevan, Julio Ponce

**Affiliations:** 1 Gastroenterology Unit, La Fe Hospital, Valencia, 21 Campanar Avenue, Valencia 46009, Spain; 2 Centro de Investigación Biomédica en Red de Enfermedades Hepáticas y Digestivas (Ciberehd), 170 Villarroel Street, escalera 12 planta 4, Barcelona 08036, Spain; 3 Coloproctology Unit, La Fe Hospital, Valencia, Campanar Avenue 21, Valencia 46009, Spain

## Abstract

**Background:**

The effect of thiopurine immunomodulators on health-related quality of life (HRQoL) in patients with inflammatory bowel disease (IBD) has been controversial. The aims were to evaluate the HRQoL in patients with IBD treated with thiopurines and assess the short- and long-term impacts of the treatment on HRQoL.

**Methods:**

Ninety-two consecutive patients who started treatment with thiopurines were prospectively included. Evaluation of HRQoL was performed at months 0, 6, and 12 using two questionnaires, the Short-Form Health Survey (SF-36) and the Inflammatory Bowel Disease Questionnaire (IBDQ).

**Results:**

Baseline score of IBDQ was 4,6, range (2,31-6,84), with an impairment of the five dimensions of HRQoL compared with inactive patients. Results obtained in 8 dimensions of SF-36 showed worse HRQoL than Spanish general population. At 6 months patients had a significant improvement in overall IBDQ score -5,8 (1,58 -6,97)- and also in all IBDQ dimensions. All the 8 dimensions of SF-36 obtained a significant improvement. At twelve months score of IBDQ was 6,1, range (2,7-6,98), with improvement in all dimensions compared with baseline and 6 months. SF-36 showed a similar significant improvement in all subscales.

**Conclusions:**

Thiopurine immunomodulators alone or with other treatments have a positive and long lasting impact on HRQoL of IBD patients.

## Background

The inflammatory bowel diseases (IBD) Crohn's disease (CD) and ulcerative colitis (UC) are chronic relapsing conditions of unknown etiology [1]. The young age of onset and the morbidity associated with these diseases and their therapies affect patients, not only physically but also through limitations in social, educational, professional and emotional activities. It is well documented that IBD patients have an impaired perception of their health-related quality of life (HRQoL) compared with an age- and sex-matched population [2-8].

Although controlling disease activity is of prime importance in patient well-being, conventional morphological measures and activity indices do not assess the subjective perception of health and the overall impact of the disease on patients' lives [9]. For these reasons, it is currently accepted that the goals of therapy should be to control symptoms, reduce complications, minimize treatment toxicity and improve quality of life [10].

The efficacy of thiopurines in the scenarios in which they are prescribed--either to induce or to maintain remission in both UC and CD--is well proven [11-14]. It is recognized that in both CD and UC, HRQoL is strongly linked to an individual's current level of symptoms [15,16]. The achievement and maintenance of remission should be accompanied by an increase in HRQoL; however, other factors could influence the effects of any treatment on HRQoL, such as patient psychosocial characteristics, outcome expectancies, fear of side effects, and attitudes and beliefs toward the treatment. Most of the useful treatments in IBD, such as mesalazine, budesonide, cyclosporine, infliximab, natalizumab, leukocyte apheresis and surgery have proven to benefit HRQoL [17-26]. The effects of immunosuppressive drugs on HRQoL have been controversial. Some authors have reported a worse HRQoL among azathioprine users while others found better HRQoL among thiopurine-treated patients [27,28]. A recent case-control study comparing the HRQoL of CD patients in stable remission with thiopurine immunomodulators with matched healthy controls showed that treatment restores HRQoL and psychological well-being to normal levels [29]. In consequence, the thiopurines may have a positive impact on HRQoL, mainly in patients with inactive disease; however, several issues should be addressed. First, information is scarce, the pattern of HRQoL of the patients before starting thiopurines is not known, and there are no published prospective studies analyzing the impact of thiopurines on different dimensions of HRQoL. Second, even less information is available in UC patients, and third, the different scenarios where thiopurines are prescribed could alter the benefits of thiopurine treatment.

Knowledge of the main affected dimensions and the effect of thiopurines on HRQoL would be of considerable help to evaluate medical treatment and quality of care, to conduct economical analysis, and to develop guidelines for clinical practice.

Therefore, the main objective of this study was to evaluate the quality of life in patients with inflammatory bowel disease treated with thiopurines and to assess the short- and long-term impacts of the treatment on different dimensions of quality of life. Additionally, we analyzed the relationships between clinical and biological variables with HRQoL.

## Methods

### Study Population

Participants were recruited from a single Spanish hospital. The diagnoses of IBD were made according to the Lennard-Jones criteria [30] and patients were classified in accordance with the Montreal classification [31]. In patients with CD, age at diagnosis, lesion location (ileum, colon, ileum and colon or upper gastrointestinal tract), disease behavior (stricturing, penetrating or nonstricturing and nonpenetrating) and the presence of perianal disease were recorded to classify the subjects. UC was classified into three subgroups: ulcerative proctitis; left-sided UC (distal UC); and extensive UC (pancolitis).

We prospectively and consecutively studied all adult subjects with IBD either attending as outpatients or during hospital admission who started treatment with thiopurines. The indications to initiate thiopurines in both CD and UC were steroid dependency, induction of remission, and maintenance treatment after a severe flare. In patients with CD, thiopurines were additionally used to treat perianal disease, to treat extensive disease, and to prevent postsurgical recurrence.

All patients who underwent surgery were endoscopically evaluated after 12 months, recurrence in the neoterminal ileum was defined by a Rutgeerts score > = 1. In case of recurrence, patients were started with Azathioprine. The patients who started thiopurines immediately after surgery were not eligible for the study.

Induction treatment with infliximab, at three doses of 5 mg/Kg, was allowed at the beginning of the study, those patients who received maintenance treatment with infliximab were excluded. The treatment was considered as failure if the patient required infliximab or surgery during the follow up.

### Treatment

All patients were treated initially with azathioprine. Although the enzyme activity of thiopurine methyltransferase (TPMT) was measured at the beginning of treatment, AZA was started at 50 mg daily and was increased to a target dose of 2.5 mg/kg within the first month of follow up [32].

### HRQoL Assessment

Prospective evaluation of HRQoL was performed at months 0, 6, and 12 using two questionnaires, the Medical Outcomes Study 36-Item Short-Form Health Survey (SF-36) and the Inflammatory Bowel Disease Questionnaire (IBDQ). The SF-36 is a generic questionnaire containing 36 items [33]. Thirty-five of the items are grouped into eight multi-item scales. The eight domains of the SF-36 are as follows: physical function (10 items); social function (two items); role limitations due to physical problems (four items); role limitations due to emotional problems (three items); energy/vitality (four items); mental health (five items); bodily pain (two items); and general health perception (five items). In addition, it contains a one-item measure of self-evaluated change in health status (health transition) over the previous year. For each question, the raw score was coded and transformed into a percentage on a scale of 0 to 100, with 0 indicating the least favorable possible health status and 100 indicating the most favorable. The questionnaire has been translated into Spanish and validated [34]. The disease-specific questionnaire used, the IBDQ, was the 36-item version by Love et al [2], a self-administered questionnaire that has been previously validated for use in Spain [35]. The 36 items are grouped into five domains of health: bowel symptoms; systemic symptoms; functional impairment; social impairment; and emotional function. Responses are scored on a 7-point Likert scale, in which 7 corresponds to the highest level of functioning. The instrument produces five dimension scores and an overall IBDQ score ranging from 1 to 7, where a higher score reflects better HRQoL.

### Assessment of Clinical Activity and Definitions of Remission

Disease activity was assessed with the Crohn's Disease Activity Index (CDAI) in patients with CD and with Mayo Clinic Score or the Disease Activity Index (CAI) in patients with UC [36,37]. Eight variables determine the CDAI score: the number of liquid stools; the extent of abdominal pain; general well-being; the occurrence of extraintestinal symptoms; the need for antidiarrheal drugs; the presence of abdominal masses; hematocrit; and body weight. In order to calculate the CDAI, the patient completed a symptom diary for the seven days before each visit. Remission was defined as defined by a CDAI score < 150. The Mayo Score consists of four items: stool frequency; rectal bleeding; findings of flexible proctosigmoidoscopy; and the Physician Global Assessment. Scores range from 0 to 12 points. Complete response (remission) is defined as complete resolution of (1) stool frequency (normal stool frequency), (2) rectal bleeding (no rectal bleeding), and (3) patient's functional assessment score (generally well). If colonoscopy was available, normal endoscopy findings were also required. The patients should be free of steroids to be considered in remission.

### Study Design and Follow up

Patients were enrolled prospectively. Demographic data were recorded during the first visit. Blood samples were taken before starting on AZA, and TPMT activity was measured. Patients were clinically evaluated at baseline, at two, four and 12 weeks, and then every three months until the end of the study. HRQoL assessment was performed at the beginning and after six and 12 months of therapy. Patients were also evaluated throughout the study period whenever they had a medical problem. At each visit, a complete physical exam was performed and patients were asked about symptoms and any systemic manifestations of the disease or its treatment. Scores of activity were calculated. Data on the use of steroids and other concomitant treatments were obtained. Those patients who discontinued their medication because of adverse events or reactions were followed in the same way as those who continued to receive therapy. In cases of treatment failure, HRQoL assessment was performed before new treatment was administered.

### Statistical Analysis

Descriptive analysis of continuous variables is presented as median and range and as counts (%) for categorical data. Data were tested for a normal distribution: the natural logarithms of variables were used to reduce the heterogeneity of variance if necessary. Repeated measures one-way ANOVA corrected with the Holm-Bonferroni method was performed to explore differences in the HRQoL values along the study. To A two-sided *P *value of less than 0.05 was considered statistically significant.

To aid interpretation and comparison, the magnitude of differences was also expressed as an effect size (the differences in scores divided by the standard deviation for the reference group) [38]. We applied the generally agreed thresholds (0.2, 0.5, and 0.8 respectively) for 'small', 'moderate' and 'large' effect sizes [39]. When the SF-36 was used, for the individual scales a change of ten or more points was considered a minimum important difference [40]. In case of IBDQ-36 questionnaire an absolute change of 0.5 points has been used to define a minimum clinically important difference [41].

To determine whether there are factors at baseline that can predict HRQoL at six months and one year, multiple regression analysis taking the global score of the IBDQ-36 as the dependent variable was performed. The analysis was performed separately for UC and CD.

## Ethical Considerations

The local research ethics committee (Comité Ético de Investigación Biomédica de Hospital Universitario "La Fe") approved the study. Investigators fully explained the details of the study, made sure that the volunteer understood the information, and allowed the patient to freely decide if they wish to participate in the study. Afterwards the patient signed the informed consent document.

## Results

### Study Patients

Ninety-two consecutive outpatients and inpatients who started thiopurine immunomodulators were prospectively included, 68 (74%) with CD and 24 (26%) with UC. All patients had a minimum follow up of six months and 74 patients had 12 months of follow up (18 patients had a follow-up period of between six and 12 months). Five and 10 patients left the study before six and 12 months, respectively. Mean TPMT levels were 19.7 (SD 4.63), range (6.6-35.7): consequently, the target dose was 2.5 mg/Kg in all patients. Twelve (13%) patients had gastrointestinal intolerance to azathioprine and in these patients; 6-mercaptopurine treatment was attempted. Of these patients, seven ceased this thiopurine treatment because of toxicity. Demographic characteristics are listed in Table 1, and the indications for treatment, disease classification, concomitant treatment and other disease-related factors are listed in Table 2. Seven patients (8%) had other comorbilities: 3 with diabetes mellitus, 3 with chronic HCV infection and one patient with mild COPD. No change was present in these diseases during the follow-up.

**Table 1:** Socio-demographic characteristics of patients included.

	Crohn's disease	Ulcerative colitis
**Number**	68	24
**Age (years)**	35 (16-70)	38 (16-67)
**Sex (male/female)**	37/31	11/13
**Smoking status**		
**Smoker**	32 (47%)	4 (16%)
**Ex-smoker**	13 (19%)	4 (16%)
**Nonsmoker**	23 (34%)	16 (67%)
**Marital status**		
**Married**	34 (50%)	6 (25%)
**Single**	29 (43%)	16 (67%)
**Separated/divorced**	5 (7%)	1 (4%)
**Widowed**	-	1 (4%)

Results are expressed as medians and range or absolute value and percentage.

**Table 2:** Clinical characteristics of patients included.

	Crohn's disease	Ulcerative colitis
**Number**	68	24
**Disease duration (months)**	27 (0-268)	11 (1-188)
**Outpatients**	61 (90%)	16 (67%)
**Previous hospitalizations**	2 (0-14)	2 (0-6)
**Previous resective surgery**	33 (49%)	-
**Localization**		
**L1: Terminal ileum**	28 (41%)	
**L2: Colon**	21 (31%)	
**L3: Ileocolon**	15 (22%)	
**L4: Upper gastrointestinal**	4 (6%)	
**E1: Ulcerative proctitis**		2 (8%)
**E2: Left-sided Ulcerative colitis**		7 (29%)
**E3: Extensive Ulcerative colitis**		15 (63%)
**Behavior**		
**B1: inflammatory**	27 (40%)	
**B2: stricturing**	26 (38%)	
**B3: penetrating**	15 (22%)	
**Perianal disease**	24 (35%)	1 (4%)
**Indication of treatment**		
**Steroid dependence**	24 (35%)	15 (62%)
**Induction of remission**	10 (15%)	5 (21%)
**Maintenance after severe flare**	1 (1%)	4 (17%)
**Fistulizing disease**	8 (12%)	-
**Prevention of recurrence**	21 (31%)	-
**Extensive disease**	4 (6%)	-
**Concomitant treatment**		
**Steroids**	35 (52%)	17 (71%)
**Infliximab**	8 (12%)	-
**Antibiotics**	18 (27%)	1 (4%)
**Mesalamine**	2 (3%)	5 (21%)
**Topical treatment**	1 (2%)	19 (79%)

Results are expressed as medians and range or absolute value and percentage.

### Basal Assessment

Basal median IBDQ score was 4.98 (range 2.31-6.88) and was higher in patients with CD (4.99, range 2.37-6.84) than in patients with UC (4.56, range 2.31-6.88), but without statistical significance. The HRQoL determined with the IBQQ showed a significant (greater than 0,5 in a 7 point likert scale) impairment of all five dimensions when compared with inactive inflammatory bowel disease Spanish patients. Results obtained for the five dimensions of the HRQoL assessed by the IBDQ and the eight dimensions analyzed by the SF-36 are shown in Table 3 for patients with CD and in Table 4 for those with UC.

**Table 3:** Health-related quality of life at baseline, six and at 12 months in Crohn's disease patients.

	Basal	6^th ^month	12^th ^month	Trend p values (*)		Differences 0 and 6^th ^month		Differences 0 and 12^th ^month	
	Mean (SD)	Mean (SD)	Mean (SD)	L	Q	Mean (CI 95%)	ES	Mean (CI 95%)	ES
**IBDQ:**									
Bowel	5,0 (0,9)	5,7 (1,1)	6,0 (1,1)	0,01	0,06	0,7 (0,4-1,0)	1,1	0,92 (0,6-1,3)	1,0
Systemic	4,4 (1,3)	5,1 (1,3)	5,4 (1,5)	0,01	0,01	0,7 (0,3-1,1)	0,5	1,0 (0,5-1,5)	0,8
Emotional	4,6 (1,2)	5,6 (1,0)	5,8 (1,2)	0,01	0,01	1,0 (0,7-1,4)	0,8	1,2 (0,8-1,6)	1,0
Social	5,1 (1,6)	6,0 (1,2)	6,2 (1,2)	0,01	0,12	0,9 (0,5- 1,3)	0,6	1,0 (0,5-1,6)	0,8
Functional	4,6 (1,5)	5,7 (1,1)	5,9 (1,3)	0.01	0.03	1,0 (0,7-1,4)	0,7	1,2 (0,7-1,7)	0,8
									
**SF-36**:									
Physical functioning	82,8 (17,4)	85,8 (16,5)	91,8 (12,4)	0,02	0,67	3,0 (-2,3- 8,3)	0,2	8,2 (3,7-12,8)	0,5
Role physical	40,2 (42,6)	63,5 (44,9)	80,6 (34,1)	0,01	0,69	23,4 (8,3-38,4)	0,6	37,8 (22,8-52,8)	0,9
Bodily pain	51,4 (25,7)	72,2 (26,9)	80,0 (22,7)	0,01	0,09	20,8 (12,5- 29,1)	0,8	30,2 (21,0- 39,4)	1.1
General health	48,2 (17,2)	53,5 (20,5)	61,3 (19,4)	0,01	0,89	5,4 (0,9-9,8)	0,3	12,8 (7,7-18,0)	0,7
Vitality	46,6 (23,9)	58,7 (24,1)	66,0 (20,2)	0,01	0,43	12,1 (4,5-19,6)	0,5	18,5 (11,2-25,8)	0,8
Social functioning	71,3 (23,6)	82,6 (20,1)	89,4 (19,9)	0,01	0,50	11,3 (4,3-18,2)	0,5	19,4 (11,3-27,6)	0,8
Role emotional	63,4 (43,3)	83,8 (33,6)	80,4 (36,1)	0,02	0,09	20,4 (8,0-32,7)	0,5	14,4 (-0,01-29,0)	0,3
Mental health	59,6 (19,7)	73,5 (16,9)	76,5 (17,6)	0,01	0,05	13,9 (8,5-19,4)	0,71	14,7 (9,6-19,9)	0,75

(*) Holm-Bonferroni corrected. L = linear, Q = quadratic, ES = effect size.

**Figure 1:**
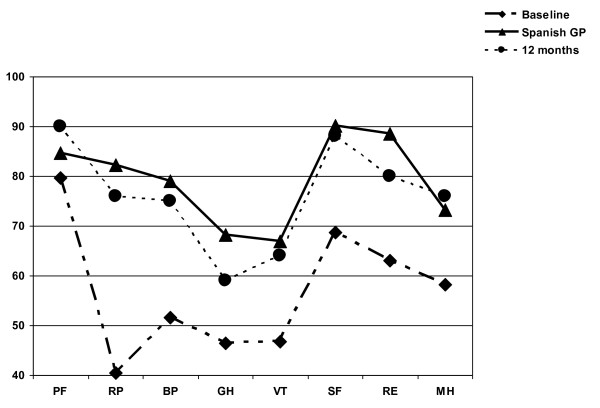
**SF-36 mean scale scores of Crohn's disease and ulcerative colitis patients in comparison to Spanish general population reference values**. **PF**: Physical Functioning, **RP**: Role physical, **BP**: Bodily Pain, **GH**: General health, **VT**: Vitality, **SF**: Social functioning, **RE**: Role emotional, **MH**: Mental health.

The HRQoL determined with the SF-36 showed a significant impairment of all eight dimensions when compared with the general Spanish population. A comparison of the SF-36 dimensions with the Spanish population is shown in Fig. 1.

The basal IBDQ scores were significantly correlated with CDAI (r = -0.56, *P *= 0.001), number of previous admissions (r = -0.25, *P *= 0.02), steroid dosage at the beginning of treatment (r = -0.50, *P *= 0.001), serum albumin (r = 0.29, *P *= 0.02) and leukocyte count (r = -0.26, *P *= 0.03). No significant correlation was seen with age, gender, duration of illness, Mayo Score index, platelet count, sedimentation rate or with fibrinogen or CRP levels. Additionally, those patients who were on steroids, hospitalized at the beginning of treatment, with the indication of induction of remission or maintenance after severe flare-up had significantly worse IBDQ scores. Other variables studied, such as the type of disease, smoking status, previous surgery or presence of perianal disease, did not have any significant influence on basal IBDQ scores. For CD patients, in the multivariate analysis, once adjusted for sex, age, duration of disease, number of previous admissions, previous surgery, presence of perianal disease and concomitant treatment at the beginning, only the CDAI and the use of steroids were associated with the basal IBDQ score. No variable was associated with the basal IBDQ score in UC patients.

#### Six-month Assessment

After six months, five of the initial 92 patients were lost to follow up, thus only 87patients were available to complete the questionnaires. At this point, 54 patients--59% as per intention to treat and 61% per protocol--were in clinical remission and free of steroids. The median global IBDQ score was 5.8 (range, 1.58-6.97). Compared with baseline, 63 (68%) patients' scores improved: the mean increase in IBDQ (ΔIBDQ) was 0.86 (SD 1.4; range -3.42 to 3.81), with an effect size of 0.68.

Differences from baseline in the five dimensions of HRQoL analyzed by the IBDQ and the eight dimensions assessed by the SF-36 are shown in Tables 3 and 4. After six months of treatment with thiopurines, all dimensions of the specific questionnaire (IBDQ) improved significantly compared with baseline. HRQoL, as assessed with the SF-36, showed a significant improvement in all dimensions with the exception of physical functioning and general health. This improvement was independent of gender, age and type of the disease except in mental health domain; in this subscale men had a significant higher score. Despite the improvement, all eight dimensions remained poorer than the reference values of the Spanish general population, as can be seen in Fig. 1.

**Table 4:** Health-related quality of life at baseline, six and at 12 months in ulcerative colitis patients.

	Basal	6^th ^month	12^th ^month	Trend p values (*)		Differences 0 and 6^th ^month		Differences 0 and 12^th ^month	
	Mean (SD)	Mean (SD)	Mean (SD)	L	Q	Mean (CI 95%)	ES	Mean (CI 95%)	ES
**IBDQ:**									
Bowel	4,6 (1,4)	5,3(1,6)	5,3 (1,3)	0,14	0,73	0,8 (0,1 - 1,5)	0,4	1,0 (-0,01 - 2,0)	0,7
Systemic	4,1 (1,5)	4,8 (1,5)	4,8 (1,2)	0,66	0,06	0,7 (-0,2 - 1,6)	0,5	0,8 (-0,1 - 1,8)	0,5
Emotional	4,5 (1,6)	5,1 (1,6)	5,3 (1,3)	0,21	0,92	0,5 (-0,3 -1,3)	0,3	0,9 (0,01 -1,7)	0,6
Social	4,1 (2,1)	5,3 (2,0)	6,0 (1,4)	0,02	0,59	1,2 (0,2- 2,3)	0,6	1,7 (0,6 - 2,9)	0,8
Functional	3,9 (1,7)	5,0 (1,8)	5,2 (1,3)	0.08	0.33	1,1 (0,2 - 2,0)	0,7	1,2 (0,3 - 2,1)	0,7
									
**SF-36**:									
Physical functioning	70,7 (25,1)	76,4 (22,6)	83,2 (18,1)	0,08	0,84	5,7 (-1,3 - 12,8)	0,2	10,4 (1,2 - 19,5)	0,4
Role physical	41,7 (44,3)	53,6 (46,3)	59,4 (38,7)	0,29	0,79	11,9 (-11,6 -35)	0,3	23,7 (-8,2 - 55,6)	0,5
Bodily pain	52,1 (28,6)	56,4 (27,8)	57,7 (20,5)	0,28	0,11	14,3 (0,6- 28,0)	0.5	10,8 (-3,4 - 25,0)	0,4
General health	41,3 (18,4)	46,0 (19,5)	50,8 (23,8)	0,14	0,60	4,7 (-3,3 - 12,6)	0,3	8,5 (-5,8 - 22,7)	0,5
Vitality	47,1 (21,2)	52,1 (25,5)	59,4 (21,8)	0,07	0,78,0,80	5,0 (-6,3 - 16,0)	0,2	16,5 (0,2 - 32,7)	0,8
Social functioning	61,3 (28,2)	73,2 (28,9)	81,6 (17,4)	0,08	0,64	11,9 (-2,1 - 26)	0,4	20,9 (2,5 - 39,2)	0,5
Role emotional	61,9 (46,3)	71,4 (38,4)	76,5 (33,1)	0,63	0,66	9,5 (-14,5 -34)	0,2	19,4 (-13,1 - 51,9)	0,4
Mental health	54,7 (22,1)	61,7 (21,9)	72,2 (20,3)	0,02		7,0 (-2,4-16,5)	0,3	15,4 (0,5 - 30,2)	0,7

(*) Holm-Bonferroni corrected. L = linear, Q = quadratic, SE = effect size.

Among the CD patients, the ΔIBDQ was significantly poorer in those patients who had had previous surgery and in those patients with the indication of prevention of recurrence. The ΔIBDQ had a good correlation with CDAI (r = -0.38, *P *= 0.002). Conversely, those patients treated with infliximab or with systemic steroids at baseline experienced a significantly greater improvement in ΔIBDQ. In UC patients, only tobacco smoking was related to the ΔIBDQ. In order to exclude confounding variables, regression analysis of clinical variables was performed. In UC patients, the ΔIBDQ was associated with tobacco smoking, and patients who smoked had small ΔIBDQs compared with never or ex-smokers. No variables were found to be associated with ΔIBDQ in CD patients.

#### Twelve-month Assessment

Seventy-four patients reached 12 months of follow up. Of them, 10 patients were lost, thus only 64 patients were able to complete the questionnaires. At this point, 45 patients--61% as per intention to treat and 70% per protocol--were in clinical remission and free of steroids. Median global IBDQ scores were 6.1 (SD 1.14; range 2.7-6.98). Compared with baseline, 47 (64%) patients' scores improved: the median ΔIBDQ was 1.05 (SD 1.28; range -2.2 to 3.7) and the effect size of the difference was 0.83.

Tables 3 and 4 give the comparisons of the IBDQ and SF-36 scores of patients at enrollment and after 12 months of treatment.

After one year of treatment with thiopurines, all the dimensions of the specific IBDQ and generic SF-36 questionnaires improved significantly compared with baseline and all the scores was higher than those obtained at six months. Only the scores of the SF-36 physical functioning and mental health were equal to the general Spanish population, with the remaining dimensions slightly worse than the reference values (Fig. 1). The gaining in SF-36 was indepedent of gender in all domains.

The ΔIBDQ at 12 months was correlated with the number of previous admissions in both CD (r = 0.58, *P *= 0.047) and UC (r = 0.30, *P *= 0.029) patients. Additionally, in those patients with CD, the improvement was significantly greater in those patients treated with steroids at the beginning of treatment. In UC patients, the gain was significantly higher in those for whom the indication of treatment was maintenance after a severe flare or induction of remission. Other clinical variables studied were not related to the ΔIBDQ. Regression analysis confirmed that the improvement in HRQoL in CD patients correlated only with steroid use at the beginning of treatment and this association was independent of the indication and other concomitant treatments. For UC patients, no association was seen in the multivariate analysis.

## Discussion

Our study shows that before initiating thiopurines, patients with IBD have greatly impaired perceived health status involving the majority of the aspects of HRQoL. Furthermore, and more importantly, this study provides evidence of the positive and long-lasting impact of azathioprine and 6-mercaptopurine on all dimensions of HRQoL in patients with IBD.

Currently, thiopurines are the cornerstone of the treatment of IBD patients and are used in several clinical situations [42]. The first issue addressed in this study was the pattern of HRQoL in patients before starting thiopurines. As expected, this group of patients had impaired HRQoL. The mean scores in the eight domains of the SF 36 were all lower than published Spanish reference data [34]. The physical function domain gave the worst scores: this dimension includes items that assess whether the patient has problems with work or other daily activities because of their physical health. It is well recognized that remission is associated with increased employment and productivity in both UC and CD [43,44]. Therefore, restoring the physical role and thus the availability to work is an important indirect endpoint.

The pattern of impaired dimensions in the IBDQ-36 showed that the bowel symptoms score was the least impaired at the beginning of treatment. Conversely, the largest impact appeared to be in the systemic domain. This pattern is different to previously published data from patients with active or inactive disease in which bowel symptoms was the worst scored domain in active disease patients and systemic symptoms the most affected domain in patients in remission [6]. These divergences can be attributed to the use of steroids and other drugs that could be able to ameliorate bowel symptoms but are not able to restore completely the quality of life of patients, probably because nonspecific symptoms such as fatigue, general discomfort, sleep disturbances and appetite problems are still present. The association of the use of steroids with a worse HRQoL in patients with CD supports this proposed explanation. Other authors have already suggested this association [45]. Recently, in a study performed with UC patients treated with infliximab, those patients who had discontinued corticosteroid use showed greater improvement in HRQoL than those who had not [22]. This interesting finding is relevant and reinforces the idea that a patient receiving steroids should not be considered in remission.

The second issue addressed, and which produced our main finding, was to provide evidence that thiopurine treatment has a positive effect on all dimensions of HRQoL. More importantly, this effect is durable, with better scores after one year of treatment than at six months. Few data are available with which to compare our results. One case-control study reported that a sustained response to thiopurine immunomodulators restores HRQoL and psychological well-being in patients with CD to values equivalent to those of healthy controls [29].

Globally, results obtained with the specific questionnaire for both CD and UC at six and 12 months showed an effect size larger than 0.5 in all dimensions studied, meaning that the magnitude of improvement in HRQoL is definitely relevant. It is important to remark that the interpretation of HRQoL scores could be complex. The interpretation could be based on the statistical significance of a change, but placing the magnitude of these changes in a context easy to understand is arduous. To provide meaningful interpretation of HRQoL intervention and treatment effects two broad strategies have been described: the effect size and the minimum clinically important difference. In our paper to facilitate the interpretation of the scores the changes are expressed in both [46]. HRQoL was slightly worse in UC than in CD patients throughout the study and the improvement seen during the study was lower in UC patients. Only the social impairment dimension (avoiding events if toilets are not nearby, canceling social engagements), the most affected difference at the beginning of treatment, showed a greater improvement in UC patients. This observation differs from previously published data because UC patients typically experience better HRQoL than those with CD [4-6]. It is difficult to speculate about this finding mainly because the available evidence suggests that thiopurine therapy is as effective in UC as it is in CD [12]. A possible explanation for our findings is that the patients with UC were all patients with severe disease, while many patients with CD were not, and those patients treated with thiopurines to prevent postsurgical recurrence and thus with inactive disease could exemplify this hypothesis.

Globally, the SF-36 showed homogeneous improvement in all dimensions, although complete restoration of HRQoL was not achieved, probably because IBD patients have a worse HRQoL despite having inactive disease. The magnitude of improvement was greater than the minimum important difference in all domains except general health at both at six and 12 months and physical functioning at six months. The effect size also showed a relevant improvement, mainly after one year of treatment. Two considerations merit comment about the SF-36 results. First, the general health dimension (considered as an indicator of patients general health) had an increase relatively small in contrast to much greater differences of many other dimensions, one explanation is that thiopurines need periodical clinical monitoring and may produce severe side effects that could decrease HRQoL. Second, there was a trend toward better results in the mental health-related domains in UC patients while CD patients obtained better results in the physical health domains. One explanation for this finding is that patients with UC had less disease duration, probably some factors that affected HRQOL could vary with the time. In contrast with this theory, some authors have published that duration of disease influences the emotional domain: the longer time with UC the better emotional well-being, suggesting a degree of adjustment over time to their condition [47].

## Conclusions

Some considerations should be taken into account before extracting conclusions. First, this is a prospective study, thus behavior of different dimensions of HRQoL during follow up can be seen. Additionally the study has a follow-up period of one year, and probably at the end of the study the initial contribution of other medications as steroids, Infliximab or Cyclosporine is not present. Additionally the length of the study allows to identify those side effects that could only be detected after the first months and that may possibly affect the HRQoL. The cohort was included prospectively and consecutively and the study was performed in clinical practice conditions, thus both CD and UC were considered along with all types of scenario where thiopurines are used. We found a significant improvement in all indications including the prevention of postoperative recurrence. This fact could be explained by several reasons. The patients were included one year after the surgery when endoscopic recurrence was established; although they were inactive at inclusion, they had symptoms that were probably affecting their HRQoL. We think that those symptoms could be caused by the endoscopic recurrence and therefore the healing of lesions induced by the azathioprine, possibly will be the cause of the improvement in the HRQoL.

Future investigations should assess the long-term impact on HRQoL of new scenarios in which thiopurines are currently used, such as early introduction in both UC and CD. In this study, we did not routinely perform endoscopy. Another important issue that should be addressed in future investigations is the implications on HRQoL and in the natural history of azathioprine-induced mucosal healing.

## Competing interests

The authors declare that they have no competing interests. The preparation of this paper was funded in part by UCB-Pharma.

## Authors' contributions

**GB **participated in its design, collected the data, performed the statistical analysis, interpreted the data and wrote the paper. **PN **jointly conceived the study with JP, participated in its design, coordinate it, interpreted the data and supervised the drafted manuscript. **MA **collected the data and gave conceptual advice. **BB **participated in its design, gave conceptual advice and participated in writing the manuscript. **MI **help during the collection of the data and participated in writing the manuscript. **VO **participated in its design and prepared the manuscript. **VG **participated in its design and supervised its statistical analysis. **RE **help during the collection of the data **JP **jointly conceived the study with PN and participated in its design. All authors read and approved the final manuscript.

## Pre-publication history

The pre-publication history for this paper can be accessed here:

http://www.biomedcentral.com/1471-230X/10/26/prepub
